# Public participation in healthcare students' education: An umbrella review

**DOI:** 10.1111/hex.13974

**Published:** 2024-01-21

**Authors:** Lorelli Nowell, Bryn Keogh, Eleftheria Laios, Lisa Mckendrick‐Calder, Whitney Lucas Molitor, Kerry Wilbur

**Affiliations:** ^1^ Faculty of Nursing University of Calgary Calgary Alberta Canada; ^2^ Communications and Media University of Calgary Calgary Alberta Canada; ^3^ Center for Teaching and Learning Queen's University Kingston Ontario Canada; ^4^ Faculty of Nursing MacEwan University Edmonton Alberta Canada; ^5^ Occupational Therapy Department University of South Dakota Vermillion South Dakota USA; ^6^ Faculty of Pharmaceutical Sciences University of British Columbia Vancouver British Columbia Canada

**Keywords:** curriculum, healthcare education, patient–public engagement, research synthesis

## Abstract

**Background:**

An often‐hidden element in healthcare students' education is the pedagogy of public involvement, yet public participation can result in deep learning for students with positive impacts on the public who participate.

**Objective:**

This article aimed to synthesize published literature reviews that described the impact of public participation in healthcare students' education.

**Search Strategy:**

We searched MEDLINE, EMBASE, ERIC, PsychINFO, CINAHL, PubMed, JBI Database of Systematic Reviews and Implementation Reports, the Cochrane Database of Systematic Reviews, Database of Abstracts of Reviews of Effects and the PROSPERO register for literature reviews on public participation in healthcare students' education.

**Inclusion Criteria:**

Reviews published in the last 10 years were included if they described patient or public participation in healthcare students' education and reported the impacts on students, the public, curricula or healthcare systems.

**Data Extraction and Synthesis:**

Data were extracted using a predesigned data extraction form and narratively synthesized.

**Main Results:**

Twenty reviews met our inclusion criteria reporting on outcomes related to students, the public, curriculum and future professional practice.

**Discussion and Conclusion:**

Our findings raise awareness of the benefits and challenges of public participation in healthcare students' education and may inform future research exploring how public participation can best be utilized in higher education.

**Patient or Public Contribution:**

This review was inspired by conversations with public healthcare consumers who saw value in public participation in healthcare students' education. Studies included involved public participants, providing a deeper understanding of the impacts of public participation in healthcare students' education.

## 1 INTRODUCTION

The pedagogy of public involvement is an approach used to engage members of the public to actively participate in decision‐making around issues that affect their lives and communities. The aim is to empower individuals and communities with the knowledge, skills and opportunities to participate in or contribute to these decisions.^1^ Emphasis is often placed on inclusivity, transparency and collaboration to foster partnerships between decision‐makers and the public.^1^, ^2^ In the context of healthcare students' education, the pedagogy of public involvement focuses on engaging patients, families and communities in teaching healthcare students.

Historically, patient involvement in teaching healthcare students has been a cornerstone of learning, where patients engage as storytellers or resources for students. However, over time this has evolved.^3^, ^4^ Recently, there has been a need to develop this involvement further towards an emerging pedagogy of public involvement throughout educational programmes.^2^, ^3^ Modigh et al.^5^ argued that this shift is a moral responsibility and that those who pay for and/or receive healthcare ought to inform how it is provided.

This emerging pedagogy holds promise to impact students, the public, educational curricula and healthcare systems. Public involvement emphasizes patient‐centred care and acknowledges the expertise and perspectives of patients and their families.^1^, ^2^, ^6^ It can promote the active involvement of patients and families in their own care, encourage shared decision‐making and foster the development of patient advocacy skills.^1^, ^4^ The pedagogy of public involvement in healthcare students' education can also enhance empathy,^7^, ^8^ communication skills,^9^ reflective practices and critical thinking that encourages students to critically examine power dynamics, social inequities and ethical considerations in healthcare. Additionally, it has benefits for patients such as empowered participation in their own care,^6^ and feeling like they have contributed to others.^10^ Integrating the principles of public involvement into healthcare education may result in more patient‐centred, community‐oriented and socially responsible healthcare systems.

The pedagogy of public participation incorporates patient and public involvement across multiple touchpoints in healthcare students' education programmes. Patient involvement range includes little involvement, emerging involvement, growing involvement, collaboration or partnership.^2^, ^4^, ^11^ While some promising evidence continues to emerge regarding the impact of public involvement in healthcare students' education, the literature lacks coherent, evidence‐based direction for how public participants, students and educators can best enhance teaching and learning experiences and ultimately improve healthcare experiences for all. Therefore, this umbrella review aimed to identify current, relevant and robust evidence on the impacts of public participation in healthcare students' education on students, public, curricula and healthcare systems.

## 2 METHODS

### 2.1 Design

In conducting our umbrella review, we followed best practices as outlined by the Centre for Reviews and Dissemination (CRD)^12^ and the Joanna Briggs Institute (JBI) guidelines for systematic reviews and research syntheses.^13^ While the JBI and CRD recommend that only systematic reviews be included in umbrella reviews, we purposefully included all types of reviews in our analysis.

### 2.2 Data sources and search strategy

An experienced librarian reviewed our literature search strategy in November 2022. We searched the following databases to identify English language reviews suitable for inclusion in this review: MEDLINE, EMBASE, ERIC, PsychINFO, CINAHL, PubMed, JBI Database of Systematic Reviews and Implementation Reports, the Cochrane Database of Systematic Reviews, Database of Abstracts of Reviews of Effects and the PROSPERO register. A complete search strategy for all included databases can be found in Table S1.

### 2.3 Eligibility criteria

Reviews were included if they:

1. focused on healthcare students, patients/public and/or healthcare educators;
2. described patient or public participation in healthcare students' education;
3. reported on the impact of patient/public participation in healthcare students' education, including impacts on students, patient/public, curricula or healthcare setting and
4. were published from 2012 onwards.

Reviews were excluded if they:

1. focused on non‐healthcare students or non‐healthcare educators;
2. did not address the patient/public participants in healthcare students' education;
3. were primary studies, commentaries, editorials or letters;
4. had not been published within the last 10 years and
5. were non‐English language reviews.

### 2.4 Selection of reviews

All search results were exported to Covidence to facilitate data management, organization and progress of this review. Studies were screened in two stages. First, study titles and abstracts were independently screened in duplicate by two reviewers. Screeners achieved an overall interrater agreement above 90% and all disagreements were resolved by a third reviewer. Second, full texts of studies included during title and abstract screening were subsequently screened in the same manner as title and abstract screening.

### 2.5 Quality appraisal

The reviews that met the inclusion criteria were assessed independently by two researchers using the Joanna Briggs Institute's Critical Appraisal Checklist for Systematic Reviews and Research Synthesis^13^ with disagreements resolved through discussion. The checklist includes 11 assessment criteria that were each scored ‘yes’, ‘no’, ‘unclear’ or ‘not applicable’. We did not exclude reviews based on their quality appraisal scores.

### 2.6 Data extraction and synthesis

One reviewer independently extracted data from the included reviews and a second reviewer reviewed the extraction data for accuracy. Any disagreements in extraction were resolved through discussion. Due to the heterogeneity of the reviews, a meta‐analysis was not possible. We instead utilized a narrative synthesis approach to describe the impact public participation in healthcare students' education has on students, the public, curricula and healthcare systems.

## 3 RESULTS

**Figure 1:**
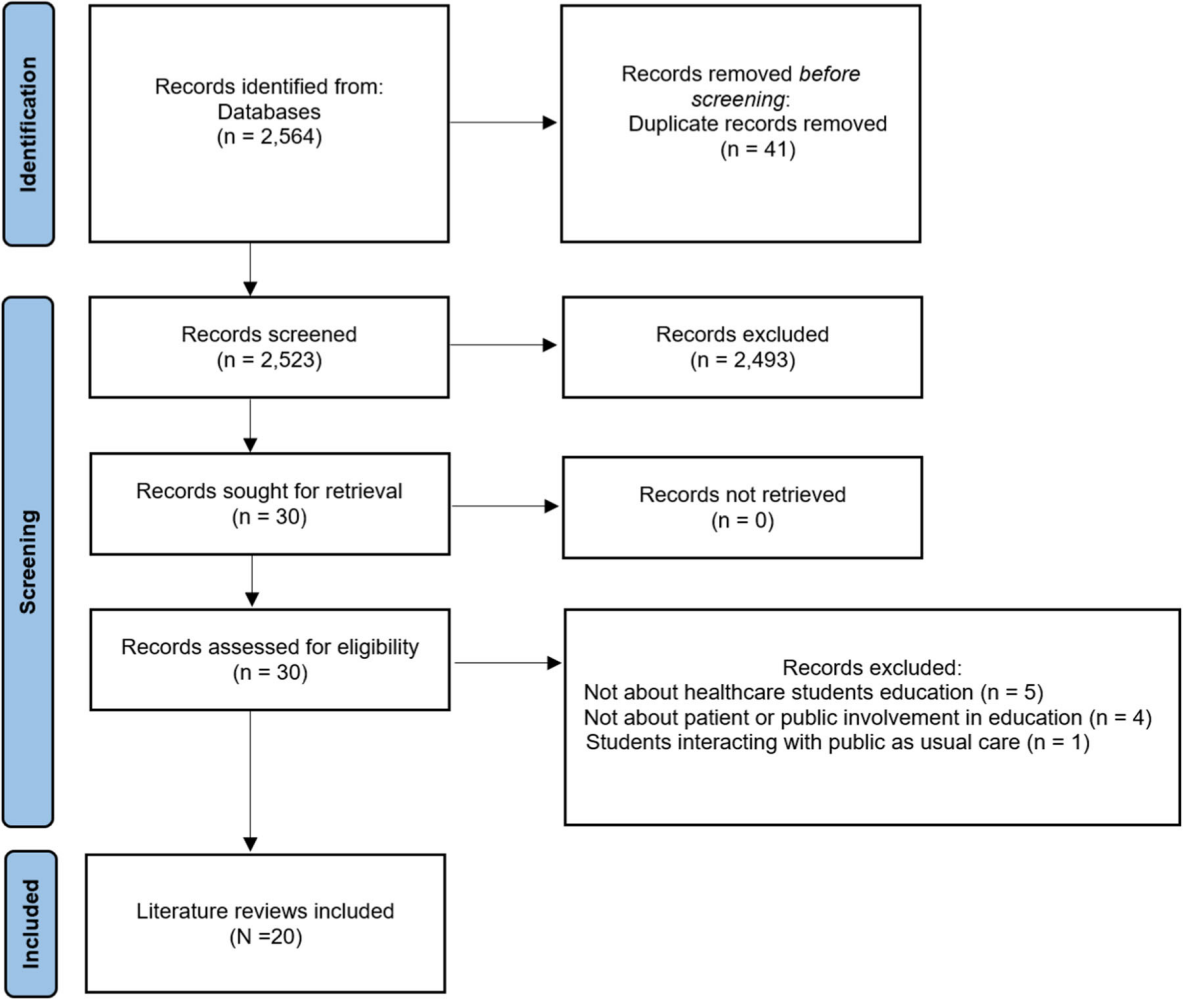
Flow of literature through the umbrella review.

Our electronic and hand searches generated 2564 records, which were screened for inclusion and 30 underwent secondary full‐text screening. Of these, 20 reviews were included in the final synthesis. Figure 1 displays the flow of literature throughout our review.

### 3.1 Review characteristics

Table 1 provides an overview of review characteristics. The included reviews were published between 2012 and 2022 with nine (45%) published since 2020. A wide range of studies were included in the reviews, with a minimum of 7 studies and a maximum of 59 studies assessed in a single review. When combined, a total of 49,014 articles were screened within the included literature reviews, and 418 were selected for synthesis.

**Table 1:** Review characteristics.

Characteristic	*N* = 20
	*n* (%)
Type of review	
Systematic review	12 (60%)
Scoping review	2 (10%)
Literature review	3 (15%)
Integrative review	2 (10%)
Theoretical systematic review	1 (5%)
Disciplines	
Multiple health and social care disciplines	6 (30%)
Medicine	7 (35%)
Nursing	6 (30%)
Pharmacy	1 (5%)
Year of publication	
2012	1 (5%)
2013	1 (5%)
2014	1 (5%)
2015	3 (15%)
2016	2 (10%)
2017	0 (0%)
2018	2 (10%)
2019	1 (5%)
2020	3 (15%)
2021	2 (10%)
2022	4 (20%)

### 3.2 Review quality

Table 2 presents the quality appraisal assessment for all included reviews. Most review articles reported sufficient information across most of the criteria; however, some authors failed to report whether the appraisal of studies included in the review was conducted independently by at least two authors; how or if data from the studies were systematically extracted, and how authors combined included studies in their results.

**Table 2:** Quality appraisal scores.

References	Review type	Review question	Inclusion criteria	Search strategy	Sources and resources	Appraisal criteria	Appraisal by two reviewers	Data extraction	Combining studies	Publication bias	Policy/practice recommendations	Future research
[32]	Literature review	Y	Y	Y	Y	Y	N	U	Y	N/A	Y	Y
[14]	Theoretical systematic review	Y	Y	Y	Y	Y	Y	U	Y	N/A	Y	Y
[15]	Systematic review	Y	Y	Y	N	Y	U	Y	Y	N/A	Y	Y
[16]	Systematic review	Y	Y	Y	Y	Y	Y	Y	U	N/A	Y	Y
[17]	Systematic review	Y	Y	Y	Y	Y	U	Y	U	N/A	Y	Y
[4]	Systematic review	Y	Y	Y	Y	Y	Y	Y	Y	N/A	Y	Y
[18]	Systematic review	Y	Y	Y	Y	N	N	U	U	N/A	Y	Y
[19]	Systematic review	Y	Y	Y	Y	Y	Y	U	Y	N/A	Y	Y
[20]	Scoping review	Y	Y	Y	Y	N	N	Y	Y	N/A	Y	Y
[21]	Integrative review	Y	Y	Y	Y	Y	Y	Y	Y	N/A	Y	Y
[22]	Integrative review	Y	Y	Y	Y	Y	U	U	U	N/A	Y	Y
[23]	Literature review	Y	Y	Y	Y	N	U	Y	U	N/A	Y	Y
[24]	Systematic review	Y	Y	Y	Y	Y	N	Y	U	N/A	Y	Y
[25]	Systematic review	Y	Y	Y	Y	Y	U	U	U	N/A	Y	Y
[26]	Systematic review	Y	Y	Y	Y	Y	Y	Y	U	N/A	Y	Y
[27]	Systematic review	Y	Y	Y	Y	Y	Y	Y	Y	N/A	Y	Y
[28]	Scoping review	Y	Y	Y	Y	Y	Y	Y	Y	N/A	Y	Y
[29]	Literature review	Y	Y	Y	Y	Y	U	Y	U	N/A	Y	Y
[30]	Systematic review	Y	Y	Y	Y	Y	Y	Y	U	N/A	Y	Y
[31]	Systematic review	Y	Y	Y	Y	Y	Y	Y	U	N/A	Y	Y

Abbreviations: N, no; N/A, not applicable; U, unclear; Y, yes.

### 3.3 Reported outcomes

Table 3 presents a high‐level overview of study characteristics, Table 4 presents items related to review findings and Table 5 presents the key themes identified within the extracted data. The following narrative synthesis presents reported outcomes related to the impact on students, the public, healthcare and curricula.

**Table 3:** Summary of review methods.

References	Review type	Guidelines	Inclusion criteria	Exclusion criteria	Search terms	Databases	Appraisal tools
[32]	Literature review	No guidelines cited	English 2012–2016 Nursing Impact of telenursing All study types	Opinion Pieces Literature reviews	Telenursing Impact Nursing	PubMed	Critical Appraisal Skills Programme (CASP)
[14]	Theoretical systematic review	STORIES^39^	English Undergraduate healthcare students in practice placements Technology for patient care	Did not include data on electronic systems used for patient care	Placement Electronic health records (EHRs)	CINAHL ERIC PubMed Scopus	Theoretical Quality Tool^40^
[15]	Systematic review	No guidelines cited	English 2008–2018 Telehealth in the medical curriculum	Focus on counselling Allied health	Graduate student Telemedicine	CINAHL ERIC PubMed PsycINFO Scopus	MMAT
[41]	Scoping review	Arskey and O'Malley^42^	English and Africans 1990 onward Medical or nursing students and/or professionals Effectiveness of live synchronous Videoconferencing based education	Allied health Other forms of online teaching Satisfaction as an outcome measure	Videoconferencing Education	CINAHL Cochrane Database of Systematic Reviews Cochrane Controlled Trial Registry Database of Abstracts of Reviews of Effectiveness EBSCOhost Medline PsycINFO PubMed SABINET	No appraisal tools mentioned
[16]	Systematic review	PRISMA	English 2010–2015 Peer‐reviewed and grey literature Mobile learning or social learning Evaluation studies and descriptive articles	Other forms of online learning without the use of mobile technologies	Undergraduate/graduate studies Educational technology Digital technology	CINAHL ERIC PubMed	McMaster University Quantitative Critical Evaluation form; Qualitative Critical Evaluation form
[17]	Systematic review	PRISMA	Training in eHealth Medical students	E‐learning other than e‐health Studies on interns, residents and doctors Not in peer‐reviewed journal Languages other than English and French No full article available	Digital health mHealth Smart heath device	Cochrane Library Medline Web of Sciences	Criteria for appraising qualitative research designed by Walsh and Downe; Medical Education Research Study Quality Instrument
[4]	Systematic review	STORIES^39^	English language 2004–2014 Delivery of telehealth‐related academic education Abstracts available	Patient education Use of telehealth Medical information education Knowledge skills and educational needs of practitioners in telehealth	Online health Education	Cochrane Library Embase ERIC PsycINFO PubMed Scopus Web of Science	Visual RAG ranking system
[18]	Systematic review	No guidelines cited	Use of EHR as an educational strategy Nursing students/faculty	No full text available Not in English	Informatics competencies Nursing education/curriculum	Academic Search Complete CINAHL Education Full Text Health Source: Nursing Academic Edition Ovid	No appraisal tools mentioned
[19]	Systematic review	PRISMA	English language 2014–2019 NP students Peer‐reviewed journals	Examination of telehealth education in undergraduate nursing	Telemedicine Nursing education	CINAHL PubMed	No appraisal tools mentioned
[20]	Scoping review	PRISMA Extension for Scoping Reviews^43^	Any language Undergraduate health‐related degree students Comparison of traditional learning and elearning/blended learning Reporting on student knowledge, student satisfaction	Traditional and complementary medicine students	Health education Web/online/virtual/computer‐assisted	Cochrane Central Register of Controlled Trials (CENTRAL) ERIC Embase Medline (OvidSP) PsycINFO (OvidSP) Web of Science	No appraisal tools mentioned
[21]	Integrative review	No guidelines cited	Full‐text articles on telehealth (using keywords)	Interventions for parents, substance use, well‐being/prevention, pregnancy and parenting themes	Education Asynchronous/synchronous technologies Clinical therapeutic relationships	APA PsycNet Embase PsycINFO PsychNET PubMed/Medline Scopus Social Sciences Citation Index Web of Science	Matrix method
[22]	Integrative review	Whittemore and Knafl^44^	2004–2019 Full text articles United States Online nursing education Faculty caring behaviours in the US	Not in English Nonnursing faculty/students Clinical education in hospitals Book chapters and unpublished doctoral work	Online education Caring/compassion/social presence	CINAHLERIC PubMed	MMAT
[23]	Literature review	No guidelines cited	Web‐based nursing education programme interventions RCTs with nonequivalent control group design	Use of simulation training and scenario‐based education	Web‐based learning Nursing student	CINAHL Cochrane Library Embase ProQuest Central PubMed RISS (domestic Korean database for dissertation and other domestic journal publications)	No appraisal tools mentioned
[24]	Systematic review	PRISMA	Systematic reviews of digital health education intervention Reviews satisfying Oxman Criteria Healthcare education Digital training Multimedia classes and online blended classes	Case studies, business reports, opinion articles Interventions with no digital mediums	E‐learning Systematic review	British Education Index Campbell Collaboration Online Library CINAHL Cochrane Database of Systematic Reviews Database of Abstract of Reviews of Effects Embase Epub ERIC Medline PROSPERO Social Science Citation Index	MMAT
[25]	Systematic review	No guidelines cited	Impact of online learning and/or blended learning Nursing students Experimental design studies, case studies, action research studies Web‐based stand‐alone educational software, computer simulation, internet discussion forums	Pilot studies Postgraduate and post registration students Simulation studies E‐learning using print‐based correspondence, video conferencing, broadcast or television or radio Teacher/instructor focused outcome	Massive Open Online Courses (MOOCs) Applications (Apps)	AUEI BREI CINAHL ERIC Medline	(CASP) Application Screening tools to reduce biases
[26]	Systematic review	PRISMA	Medical undergraduate students Online learning compared with offline learning/classroom teaching Comparison with mean score and standard deviations of tests and control groups available	Postgraduates and professionals No comparison of online and offline teaching	Online learning Medical education	Embase Medline PubMed Scopus Web of Science	Medical Education Research Study Quality Instrument
[45]	Meta‐narrative review	RAMESES guidelines	Education/training of EMR Evaluating education and training results Healthcare professionals or students Specified outcomes	Review papers, reports, editorials, letters to the editor, dissertations and commentaries No healthcare students or professionals, no healthcare information technology	Education EHR	CENTRAL ERIC PubMed Scopus Web of Science	No appraisal tools mentioned
[27]	Systematic review	PRISMA	Student learning outcomes Telehealth/telemedicine outcomes and experiences	No student outcomes	Telehealth Occupational therapy Student	Academic Search Complete CINAHL Cochrane ERIC OTseeker ProQuest Central PsycINFO PubMed	CASP^46^ and Moule et al.^47^
[48]	Scoping review	PRISMA Extension for Scoping Reviews^43^	French or English Available at UofT Library System Telemedicine care curricula for physicians or physicians in training	Reviews Clinical telemedicine programme without educational components Personal experiences with telemedicine and no curriculum details	Education Curriculum Telemedicine	Embase ERIC Medline PsycINFO	No appraisal tools mentioned
[28]	Scoping review	No guidelines cited	English Before 2014 Psychiatry residents Real‐time videoconferencing technology for direct and indirect care	Other levels of training Use of technology for distance learning Not in English	Telehealth Medical education	CINAHL Cochrane Embase ERIC Medline PsycINFO	Reilly et al.^49^
[29]	Literature review	No guidelines cited	Portuguese, English, Spanish 2009–2019 Original articles, reviews, case studies, letters to the editor and editorials Nurses from Latin America and the Caribbean	Duplicate reports	Telenursing Education	BIREME SciELO	University of York NHS Centre for Reviews and Dissemination,^50^ Greenhalgh^51^
[30]	Systematic review	PRISMA	Implementation/evaluation of telemedicine‐related curriculum Undergraduate medical education	Older than 2009 No examination of evaluating telemedicine curriculum	Telemedicine Undergraduate medical education	Scopus	MERSQI
[31]	Systematic review	PRISMA					MMAT^52^

Abbreviations: EMR, Electronic Medical Records; PRISMA, Preferred Reporting Items for Systematic Reviews and Meta‐Analyses; RAG, red, amber, green.

**Table 4:** Summary of review findings.

References	Discipline	Review question/aim	# studies reviewed	# studies included	Key findings
[32]	Health and social care disciplines	1. What evidence exists to guide mental health consumer participation at each stage of the education process? 2. What evidence exists to support the effectiveness of consumer participation in mental health education in producing graduates with recovery‐oriented practice capabilities?	2340	36	Impact on students: Increased awareness and understanding of mental health issues Improved empathy and attitudes towards individuals with mental health challenges Enhanced communication and interpersonal skills Greater self‐reflection and personal growth Heightened awareness of the social determinants of mental health Elevated confidence in providing support to individuals with mental health challenges Enriched ability to address stigma and discrimination Impact on patients/public/consumers: Increased empowerment and active involvement in their own care Improved trust and rapport with healthcare providers Enhanced shared decision‐making and collaborative care Reduced stigma and discrimination towards individuals with mental health challenges Greater understanding and recognition of recovery‐oriented practices Improved mental health outcomes and quality of life Potential future impact on healthcare: Improved collaboration and partnerships between mental health consumers and healthcare professionals Enhanced service planning and delivery based on consumer feedback and preferences Increased effectiveness of mental health services through consumer participation Greater accountability and transparency in mental health systems Improved quality of care and patient satisfaction Enhanced policy development and implementation based on consumer perspectives Reduced healthcare disparities and improved access to mental health services Considerations for curriculum: Integration of consumer perspectives and experiences into educational programmes Inclusion of recovery‐oriented principles and practices in mental health curricula Emphasis on person‐centred care and shared decision‐making Enhanced focus on cultural competence and addressing health disparities Incorporation of consumer‐led initiatives and advocacy into the curriculum Integration of real‐life case studies and consumer narratives into teaching materials
[14]	Health and social care disciplines	1. How is theory framed and applied to patient involvement in teaching and learning in professional health and social care education? 2. How is theory framed and applied to the integration of patients into an educational community and their involvement in the faculty?	4848	7	Impact on students: Increased understanding of patient perspectives and experiences Enhanced communication and interpersonal skills Improved empathy and patient‐centredness Increased awareness of social determinants of health Improved critical thinking and problem‐solving abilities Increased motivation and engagement in learning Enhanced cultural competence and sensitivity to diverse patient needs Impact on patients/public/consumers: Improved patient satisfaction and trust in healthcare providers Enhanced patient safety and quality of care Increased patient empowerment and shared decision‐making Improved health outcomes through better adherence and self‐management Potential future impact on healthcare: Improved patient–provider communication and relationships Increased patient involvement in healthcare decision‐making Enhanced collaboration and partnership between healthcare providers Improved healthcare outcomes and patient safety Increased healthcare system responsiveness and patient‐centeredness Consideration for curriculum: Integration of patient perspectives and lived experiences into educational materials Development of patient‐centred teaching strategies and methods Increased emphasis on interprofessional collaboration and teamwork Enhanced focus on communication and relationship‐building skills Inclusion of patient feedback and evaluation in curriculum development
[15]	Medicine	To identify the scope of active patient involvement in medical education, the current knowledge gaps relating to rationale and motivation for involvement, recruitment and preparation, roles, learning outcomes and key procedural contributors.	769	49	Impact on students: Increased engagement and motivation Enhanced communication and interpersonal skills Improved understanding of patient perspectives and experiences Increased confidence in clinical decision‐making Development of teamwork and collaboration skills Impact on patients/public/consumers: Improved patient satisfaction and experiences Increased trust and collaboration between patients and healthcare providers Better adherence to treatment plans and shared decision‐making Enhanced patient safety Empowerment of patients in managing their own healthcare Potential future impact on healthcare: Enhanced quality of care through improved patient–provider relationships Promotion of patient safety and reduction in medical errors Increased patient engagement and shared decision‐making Promotion of patient‐centred care as a standard practice Development of healthcare professionals who are responsive to patient needs and preferences. Considerations for curriculum: Integration of patient perspectives and feedback into curriculum design Promotion of patient‐centred care as a core principle Increased emphasis on communication and interpersonal skills training Development of cultural competency and understanding of diverse patient populations Incorporation of real‐life clinical scenarios and patient narratives in teaching
[16]	Medicine	How does feedback from patients impact healthcare student's clinical skill development and learning?	164	12	Impact on students: Improved self‐awareness and self‐reflection skills Enhanced clinical skills development and learning Increased engagement in learning Development of communication and interpersonal skills Better understanding of patient‐centred care and empathy Impact on patients/public/consumers: Enhanced patient satisfaction and experiences Improved patient–provider communication and collaboration Increased trust and confidence in healthcare professionals Promotion of patient empowerment and shared decision‐making Potential for better health outcomes and adherence to treatment plans Potential future impact on healthcare: Improved quality of care through patient‐centred approaches Increased patient safety Enhancement of patient–provider relationships and trust Promotion of continuous improvement and reflective practice amongst healthcare professionals Potential for better health outcomes and overall healthcare system performance Considerations for curriculum: Integration of patient feedback as a valuable component of the curriculum Emphasis on patient‐centred care and communication skills training Incorporation of real‐life clinical scenarios and patient interactions in teaching Development of feedback skills amongst students to elicit and utilize patient perspectives
[17]	Health and social care disciplines	What is reported in the literature regarding children and adolescents who work as standardized patients in health professional education?	58	15	Impact on students: Improved communication and interpersonal skills with children and adolescents Increased confidence in interacting with paediatric patients Enhanced understanding of child development and behaviour Development of empathy for patients and peers Improved clinical decision‐making skills specific to paediatric cases Impact on patients/public/consumers: Enhanced patient experiences and satisfaction amongst children and adolescents Increased trust and comfort in healthcare interactions with students Improved communication and engagement between healthcare providers and paediatric patients Promotion of child‐centred and family‐centred care approaches Reduced unnecessary procedures Potential future impact on healthcare Improved quality of care for paediatric patients Reduced medical errors and improved patient safety Enhanced collaboration and teamwork amongst healthcare providers in paediatric settings Promotion of evidence‐based practices specific to children and adolescents Development of healthcare professionals who are skilled in paediatric care and meet the unique needs of this population Considerations for curriculum: Integration of children and adolescents as simulated patients in educational activities Promotion of paediatric‐specific clinical skills training Enhancement of paediatric‐focused communication and assessment skills Incorporation of child development and psychosocial aspects into curriculum
[4]	Medicine	1. What service user involvement is taking place in medical education? 2. To what extent does this involvement impact student's education? 3. How and why is learning impacted by service user involvement?	6155	39	Impact on students: Increased understanding of patient perspectives and experiences Enhanced communication and interpersonal skills Improved empathy and patient‐centred care approach Development of teamwork and collaboration skills Increased awareness of psychological, emotional, social, and cultural determinants of health Impact on patients/public/consumers: Enhanced patient satisfaction and experiences Increased trust and collaboration between patients and healthcare providers Promotion of patient empowerment and shared decision‐making Improved patient safety through active involvement in clinical education Potential for better health outcomes and adherence to treatment plans Potential future impact on healthcare: Improved quality of care through patient‐centred approaches Increased patient safety and reduced medical errors Promotion of patient engagement and shared decision‐making Development of healthcare professionals who are responsive to patient needs and preferences Enhancement of the patient–provider relationship and trust Considerations for curriculum: Integration of patient perspectives and feedback into curriculum design Promotion of patient‐centered care as a core principle Incorporation of patient narratives and experiences in teaching Development of cultural competency and understanding of diverse patient populations Enhancement of communication and relationship‐building skills Incorporating patients as teachers or tutors
[18]	Health and social care disciplines	What is the state of evidence for consumer involvement in the tertiary‐level education of psychiatrists, nurses, psychologists, social workers and occupational therapists?	487	20	Impact on students: Increased understanding and empathy towards the experiences of mental health service users Improved communication and interpersonal skills specific to working with mental health consumers Enhanced cultural competence and awareness of diverse perspectives Development of person‐centred care approaches Increased knowledge and skills in recovery‐oriented practices Impact on patients/public/consumers: Enhanced patient experiences and satisfaction in mental health services Increased collaboration and partnership between mental health professionals and service users Promotion of shared decision‐making and self‐determination in treatment planning Empowerment of mental health consumers in their own care Reduction in stigma and discrimination through improved understanding and interaction Potential future impact on healthcare: Improved quality of mental health services through person‐centred and recovery‐oriented approaches Increased engagement and satisfaction amongst mental health consumers Reduction in treatment disparities and increased access to appropriate care Promotion of recovery and well‐being outcomes for mental health service users Development of mental health professionals who are responsive to consumer needs and preferences Considerations for curriculum: Integration of mental health consumer perspectives into curriculum design Promotion of recovery‐oriented principles as a core component of education Incorporation of lived experience narratives and consumer‐led teaching methods Development of skills in trauma‐informed care and cultural sensitivity Enhancement of interprofessional collaboration and teamwork skills
[19]	Medicine	To systematically review published work that has explored terminally ill patient's views about being involved in undergraduate medical teaching	1540	7	Impact on students: Increased understanding of the experiences and perspectives of terminally ill patients Improved communication and empathy skills specific to end‐of‐life care Enhanced ability to provide compassionate and patient‐centred care Development of skills in discussing difficult topics and delivering sensitive information Increased awareness of the psychosocial and emotional aspects of end‐of‐life care Impact on patients/public/consumers: Empowerment of terminally ill patients in sharing their experiences and contributing to medical education Increased trust and confidence in healthcare professionals through student engagement Enhanced communication and relationship‐building between patients and healthcare providers Potential for improved end‐of‐life care and support Promotion of patient advocacy and shared decision‐making Potential future impact on healthcare: Improved quality of end‐of‐life care through increased student exposure and education Promotion of patient‐centred and compassionate approaches in terminal illness management Enhanced communication and collaboration between healthcare providers and terminally ill patients Development of healthcare professionals who are sensitive to the needs and preferences of patients at the end of life Potential for reduced disparities and improved access to palliative and hospice care services Considerations for curriculum: Integration of terminally ill patients' perspectives into curriculum design Promotion of patient‐centred and palliative care as essential components of medical education Development of skills in discussing end‐of‐life care options and goals of treatment Incorporation of ethical considerations and decision‐making frameworks in terminal illness Enhancement of cultural competency and understanding of diverse beliefs and practices related to death and dying
[20]	Medicine	1. What is the extent, nature and range of literature that exists exploring patient involvement in the assessment of postgraduate medical learners? 2. What factors appear to influence (e.g., affordances and barriers) patient involvement in competency‐based assessment?	821	41	Impact on students: Enhanced understanding of patient perspectives and experiences in their own learning and assessment Improved communication and interpersonal skills through interactions with patients Increased awareness of patient‐centred care and the importance of patient input in assessment Development of empathy and professionalism in working with patients Promotion of reflective practice and self‐assessment skills Impact on patients/public/consumers: Empowerment of patients in contributing to the assessment process and shaping the education of future healthcare professionals Increased trust and confidence in healthcare providers knowing that their perspectives are valued in assessment Improved patient–provider communication and collaboration through student–patient interactions Potential for better quality and patient‐centred care as a result of the feedback loop between patients and learners Promotion of patient advocacy and shared decision‐making in healthcare Potential future impact on healthcare: Improved quality of healthcare through the inclusion of patient perspectives in the assessment of learners Enhanced patient safety by fostering a culture of continuous learning and improvement Increased patient satisfaction and experiences through the involvement of patients in shaping future healthcare professionals Promotion of patient‐centred care as a standard practice in healthcare settings Development of healthcare professionals who are responsive to patient needs and preferences. Considerations for curriculum: Integration of patient involvement in assessment methods and processes Development of patient‐centred assessment criteria and tools Enhancement of communication and interpersonal skills training in the curriculum Incorporation of patient feedback and perspectives in curriculum evaluation and improvement Promotion of professionalism and patient‐centred care as core values in medical education
[21]	Nursing	1. What were the attributes of consumer involvement in mental health nursing education in the last 10 years? 2. What were the outcomes of consumer involvement in mental health nursing education for nursing students in the last 10 years?	1736	14	Impact on students: Improved understanding of consumers' perspectives and experiences with a positive change in beliefs and attitudes Increased empathy and compassion towards mental health consumers (reduction of negative stereotypes, seeing patients holistically) Enhanced communication and therapeutic skills Some reported an increase in anxiety or worry Potential future impact on healthcare: Enhanced awareness of equality, fairness and partnership Considerations for curriculum: Integration of consumer perspectives into educational content enhanced the education experience (student perception) Incorporation of consumer narratives and experiences in teaching materials Emphasis on recovery‐oriented care and shared decision‐making Strengthening of collaboration between academia and mental health services Curriculum ought to be well planned and scaffolded and ought to avoid further stereotyping of negative viewpoints
[22]	Nursing	1. What types of activities are implemented in service learning for nursing students? 2. What educational and noneducational benefits does the use of service learning methodology deliver to nursing students? 3. What are students' perceptions who take part in the service learning activities?	1782	22	Impact on students: Increased empathy and understanding towards patients/public/consumers Reduces negative attitudes towards patients Enhanced communication, interpersonal, skills and teamwork Increased knowledge of the community and commitment to helping the community Facilitated application of knowledge to practice Increased self‐confidence Development of cultural competence and awareness Potential future impact on healthcare: Strengthened partnerships between academia and healthcare organizations Increased community engagement and awareness of healthcare needs Promotion of a holistic approach to healthcare delivery Considerations for curriculum: Integration of service learning components into nursing education Emphasis on community‐based care and population health Incorporation of real‐world experiences into classroom learning Promotion of interdisciplinary collaboration and teamwork skills
[23]	Medicine	To understand how participation in student‐run clinics influences the professional development of medical students and elucidate benefits for patients and students	10,201	7	Impact on students: Increased positive attitude towards the care of underserved patients Increased consideration of pursuing primary care work upon graduation Impact on patients/public/consumers: Increased patient satisfaction Enhanced listening skills Potential future impact on healthcare: Enhanced understanding of caring for diverse patient populations Considerations for curriculum: Integration of student‐run clinic experiences into medical education Provided opportunities for practical application of classroom learning in a real‐world setting
[24]	Pharmacy	To explore the evidence relating to the involvement of patients in the education of student pharmacists, in terms of the nature, extent and outcomes of their contribution	5369	12	Impact on students: Decreased stigma towards mental illness Enhanced attitudes and self‐reported practice skills Improved communication and counselling skills Increased confidence in interacting with patients Development of empathy and patient‐centred approach Impact on patients/public/consumers: Improved understanding of medication use and adherence Increased empowerment, confidence and communication skills Increased satisfaction from sharing knowledge and experiences Potential future impact on healthcare: Improvement in future healthcare practice Considerations for curriculum: Integration of patient interactions into pharmacy education Practical application of knowledge and skills in a real‐world setting
[25]	Health and social care disciplines	1. What can the current evidence base reveal about the effects of mental health service user involvement in the teaching of interpersonal skills to mental health students? 2. What are the effects of this type of involvement in comparison with more traditional methods of teaching? 3. What were the participants' experiences of this type of involvement? 4. Does mental health service user involvement in the teaching of interpersonal skills to mental health students have any negative effects?	1233	10	Impact on students: Increased understanding of service users' perspectives and experiences Enhanced empathy and sensitivity towards patients/public/consumers Enhanced holistic view of patient Increased appreciation for partnership Improved communication and interpersonal skills Development of collaborative and person‐centred care approach Potential future impact on healthcare: Increased awareness of power imbalance and the need for partnership in practice Considerations for curriculum: Increased cognitive dissonance and understanding of feelings of powerlessness that service users have Report of student viewing service users as unqualified to teach and not as good as learning from teachers Important not to over‐emphasize a single experience when using service user involvement
[26]	Health and social care disciplines	To synthesize learning outcomes that result from the involvement of patients in the nutrition of dietetic student education and to consider if these interactions promote patient‐centred care	6459	13	Impact on students: Improved understanding of patients' perspectives and experiences Enhanced communication and interview/counselling skills Increased confidence in clinical skills, self‐reflection and professionalism Development practice skills in malnutrition screening and referral Impact on patients/public/consumers: Increased satisfaction with services received Enhanced positive health outcomes
[27]	Nursing	To synthesize published literature on service user involvement in undergraduate nursing education and examine how students are exposed to engagement with service users	279	11	Impact on students: Increased critical reflection Gained greater awareness of the patients' perspectives Enhanced authentic connections and deep learning Impact on patients/public/consumers: Enhanced knowledge development. Patients felt used versus valued for their participation Considerations for curriculum: Increased opportunities for real‐world learning Challenges with logistics and resources Include patients in the planning process. Perception amongst faculty that the experiences enhanced the educational experience Strengthened partnerships between the programme and the public Added insights into the admissions selection process of nursing students when the public was involved
[28]	Nursing	1. To review and summarize the existing empirical literature regarding patients' involvement in nursing students' clinical education with a focus on patients' perspective in clinical placements 2. What is the degree of patient initiative in clinical education? 3. What are the determinants of patient involvement in clinical education?	3594	32	Impact on students: Increased relational bonds Enhanced learning through the sharing of knowledge and experiences Impact on patients/public/consumers: Increased relational bonds. Strengthen feelings of being respected and valued Felt positive regarding giving feedback to students, although some patients struggled to provide critical feedback
[29]	Nursing	1. To explore the nature of service user involvement in the classroom in preregistration mental health nurse education 2. To gain insight into the prerequisites and process needed to prepare for service user involvement in the classroom 3. To consider the reported outcomes of service user involvement for student learning 4. To examine the evidence of student engagement with this type of teaching and learning	708	8	Impact on students: Increased learning not available in textbooks Gained empathy and knowledge Demonstrated patient‐centeredness Enhanced listening and questioning skills Gained insight into the reality of the patients' experiences Increased insight about ideas for self‐improvement Impact on patients/public/consumers: Increased confidence, empowerment and encouragement Enhanced opportunities to express feelings about care delivery in prior experiences Challenged stereotypes Potential future impact on healthcare: Enabled the ‘them and us’ culture to be addressed Positively impact future care provided by students Increased patient‐centred awareness and practice
[30]	Medicine	1. To identify and evaluate studies that assessed patients' attitudes towards medical student participation 2. Does patient acceptance of medical student participation vary across specialities? 3. What are patients' reasons for accepting and refusing medical student participation?	631	59	Impact on patients/public/consumers: Increased desire to contribute to the education of others Perceived a higher quality of care Increase in knowledge Improved self‐esteem when helping learners
[31]	Nursing	To provide an accurate overview of community‐based education on undergraduate nursing student skills	90	17	Impact on students: Enhanced professional skills Increased communication skills Enhanced self‐confidence Improved critical thinking skills

**Table 5:** Summary of impact on students, impact on patients, potential impact on healthcare and considerations for curriculum.

	Impact on students						Impact on patients						Potential impact on healthcare						Considerations for curriculum								
References	Increased awareness and understanding of patients' experiences	Greater empathy	Enhanced communication and interpersonal skills	Self‐reflection and personal growth	Increased confidence in supporting patients	Greater ability to address stigma and discrimination	Empowerment to be more active in care	Increased trust in healthcare providers	Shared decision making and collaborative care	Decreased stigma and discrimination	Enhanced understanding of recovery	Increased mental health and quality of life	Collaboration between patients and healthcare providers	Better healthcare service based on consumer participation	Increased accountability and transparency	Better care and increased satisfaction	Consumer‐informed healthcare policies	Decreased healthcare disparities	Integrating patient perspectives into education	Embedding consumer‐led initiatives into the curriculum	Incorporating real‐life case studies into learning	Utilizing patient‐ centred teaching strategies	Promoting patient advocacy and professionalism	Incorporating patients as teachers	Including recovery‐oriented practices	Enhancing cultural competence	Increasing communication and teamwork
[32])	X	X	X	X	X	X	X	X	X	X	X	X	X	X	X	X	X	X	X	X	X		X		X		
[14]	X	X	X			X		X	X				X						X			X					X
[15]	X		X		X		X	X	X					X		X	X	X	X		X	X				X	X
[16]	X	X	X	X			X	X	X				X	X		X		X	X	X	X	X					
[17]		X	X		X			X	X							X											
[4]	X	X	X				X	X	X		X		X	X		X			X			X		X		X	X
[18]	X	X	X			X	X		X	X				X		X		X	X		X				X	X	X
[19]	X	X	X				X	X					X	X				X	X			X				X	
[20]	X	X	X	X			X	X	X				X	X		X	X		X			X	X				X
[21]		X	X			X				X							X		X							X	X
[22]	X	X	X		X	X															X						X
[23]	X	X				X		X								X					X						
[24]		X	X		X	X	X									X	X				X						
[25]	X	X	X										X														
[26]	X		X		X											X											
[27]	X			X				X			X						X										
[28]	X						X	X	X																		
[29]	X	X	X		X		X			X		X					X										
[30]									X	X																	
[31]	X		X		X																						

### 3.4 Impact on students

Of the 20 articles included in this review, all reported the impacts of public participation in healthcare students' education on students.

#### 3.4.1 Increased awareness of patient's perspectives and experiences

Sixteen articles reported students developed an increased understanding of patient's perspectives and experiences by engaging with the public through their education.^4^, ^14^, ^15^, ^16^, ^18^, ^19^, ^20^, ^22^, ^23^, ^25^, ^26^, ^27^, ^28^, ^29^, ^31^, ^32^ For some, this awareness led to a reduction in the stigmatization of certain conditions such as mental illness. As a result of interacting with the public, students reported an increase in their awareness of patients' experiences with a variety of medical conditions. Interestingly, no articles reported on unconscious bias.

#### 3.4.2 Greater empathy

Fourteen articles reported that students developed greater empathy for patients through their interactions with the public.^4^, ^14^, ^16^, ^17^, ^18^, ^19^, ^20^, ^21^, ^22^, ^23^, ^24^, ^25^, ^29^, ^32^ This included greater understanding and empathy for medical, social and cultural factors that often impact overall health and wellbeing.^22^, ^23^, ^24^, ^32^ By speaking with patients about their perspectives and experiences, students developed a greater awareness and empathy for a variety of challenges patients may face.

#### 3.4.3 Enhanced communication and interpersonal skills

Enhanced communication and interpersonal skills were reported in 16 articles.^4^, ^14^, ^15^, ^16^, ^17^, ^18^, ^19^, ^20^, ^21^, ^22^, ^24^, ^25^, ^26^, ^29^, ^31^, ^32^ Students demonstrated improved communication skills and stronger self‐confidence and critical thinking when communicating with patients.^31^ Some students found they communicated using less professional jargon^28^ or they more easily discussed difficult topics or sensitive information.^18^

#### 3.4.4 Self‐reflection and personal growth

Student self‐reflection and personal growth were identified in four articles as impacts of public participation in healthcare students' education.^16^, ^20^, ^27^, ^32^ Khalife et al.^20^ explained that self‐reflection following interactions with the public allowed healthcare students to accept patients' needs and improve as practitioners. Scammell et al.^27^ identified that patient interactions followed by self‐reflection gave healthcare students better insights into their goals and future careers.

#### 3.4.5 Increased confidence in supporting patients

Eight articles discussed the impacts of public involvement in healthcare students' education on developing students' confidence to support patients.^15^, ^17^, ^22^, ^24^, ^26^, ^29^, ^31^, ^32^ When given the opportunity to work with members of the public, students utilized skills they learned throughout their education in real‐world settings. This increased their overall confidence in their ability to provide competent and supportive care to serve the needs of their patients.^14^

#### 3.4.6 Greater ability to address stigma and discrimination

Seven articles identified that stigma and discrimination are present in healthcare settings and public involvement in healthcare students' education is one way to address it.^14^, ^18^, ^21^, ^22^, ^23^, ^24^, ^32^ Arblaster et al.^32^ discussed the many forms of stigma people face, such as mental health issues, poor or unequal living circumstances and adversity to recovery, and suggested students can address stigma and discrimination by supporting social inclusion, challenging stigmatizing attitudes, promoting positive understandings of peoples struggles and partnering with communities. Community engagement and exposure to the patients' struggles provide students with some knowledge and experiences to address stigma and discrimination.

### 3.5 Impact on the public

Sixteen of the 20 articles reported outcomes of the impact of public participation in healthcare students’ education on the public.

#### 3.5.1 Empowered to be more active in care

Ten articles reported that when the public participated in healthcare students' education, they became more actively involved in their own care.^4^, ^15^, ^16^, ^18^, ^19^, ^20^, ^24^, ^28^, ^29^, ^32^  Engaging with healthcare students resulted in the public developing an increased sense of empowerment to be more involved in making medical decisions.

#### 3.5.2 Increased trust in healthcare providers

Eleven articles reported that when the public interacted with healthcare students it resulted in increased trust between the public and healthcare providers.^4^, ^14^, ^15^, ^16^, ^17^, ^19^, ^20^, ^23^, ^27^, ^28^, ^32^ These interactions also improved patient satisfaction^4^, ^14^, ^15^, ^16^, ^17^, ^23^, ^26^, ^27^ and improved communication between healthcare providers and the public.^16^, ^17^, ^19^, ^20^

#### 3.5.3 Shared decision‐making and collaborative care

Ten articles reported that public engagement in healthcare students' education resulted in shared decision‐making and more collaborative care.^4^, ^14^, ^15^, ^16^, ^17^, ^18^, ^20^, ^28^, ^30^, ^32^ Shared decision‐making can empower patients when they are consulted within their care.^16^ Arblaster et al.^32^ explained that public consumers of healthcare feel supported and empowered by collaborative care approaches and being involved in students' education.

#### 3.5.4 Decreased stigma and discrimination

Five articles reported on how public participation in healthcare students' education impacted public experiences of stigma and discrimination.^18^, ^21^, ^29^, ^30^, ^32^  The decline of stigma and discrimination was described previously regarding its impact on students; however, this was also identified as an impact on the public. Arblaster et al.^32^ acknowledged that the most common outcome of consumer participation in healthcare education was a decrease in stigma. Public involvement in education actively decreased the stigma and discrimination against members of the public because healthcare students learned how to consider patient perspectives during their education.

#### 3.5.5 Enhanced understanding of recovery

Three articles reported that when patients interacted with healthcare students, patients developed an enhanced understanding of their recovery.^4^, ^27^, ^32^ Public participation in healthcare students’ education contributed to moving to recovery‐orientated practice specifically for those with mental health concerns.^32^

#### 3.5.6 Increased mental health and quality of life

Two articles reported on how patient participation in healthcare students' education resulted in patients having an increased sense of mental health and quality of life.^29^, ^32^  Mental health needs to be more readily addressed within healthcare, and public involvement in education may be one way to address this need. When students developed skills to hear and respond to patients and their experiences, patients felt well taken care of and not discouraged or hindered by their mental health. However, these benefits were not universal. Terry^28^ reported that patient engagement in healthcare students' education was stressful.^28^

### 3.6 Potential impact on healthcare

Sixteen out of 20 reviews included in this study highlighted the potential impact that public participation in healthcare students’ education has on healthcare more broadly than just on individual patients and students.

#### 3.6.1 Collaboration between patients and healthcare providers

Seven articles reported on the potential of public participation in healthcare students’ education to increase collaboration between patients and healthcare providers more broadly.^4^, ^14^, ^16^, ^19^, ^20^, ^25^, ^32^ Gordon et al.^4^ found that the collaboration between patients and healthcare providers had the potential to improve healthcare overall.

#### 3.6.2 Better healthcare service based on consumer participation

Seven articles reported that public participation in healthcare students' education resulted in better healthcare services created based on consumer participation.^4^, ^15^, ^16^, ^18^, ^19^, ^20^, ^32^ Patient views of the impact of being involved in the education of healthcare students included personal fulfilment in the belief they were improving the healthcare system.^14^ Others reported the involvement of patients led to improved outcomes^15^ and an increased understanding of patient‐centred^4^ holistic care,^32^ potentially impacting healthcare services.

#### 3.6.3 Increased accountability and transparency

The theme of accountability and transparency was present in one article. Arblaster et al.^32^ took a comprehensive view regarding accountability and transparency within a patient‐worker collaborative model and found where patients were teaching and advising on their health to students it allowed for younger patients to be heard by those who are usually in a place of power. When patients are more involved in their care, healthcare providers can have a higher sense of accountability to ensure they share knowledge and account for the patient's perspective.^32^

#### 3.6.4 Better care and increased satisfaction

Ten articles reported that when the public participated in healthcare students’ education, they received better care, which resulted in increased satisfaction.^4^, ^15^, ^16^, ^17^, ^18^, ^20^, ^23^, ^24^, ^26^, ^32^ When patients participated in teaching, healthcare providers learned patient‐centred care and holistic care. The overall increase in patient satisfaction with their care allows greater confidence in healthcare systems potentially making healthcare better all around.

#### 3.6.5 Consumer‐informed healthcare policies

Eight articles reported that when the public participates in healthcare students' education, this can potentially lead to more consumer‐informed healthcare policies.^15^, ^20^, ^21^, ^24^, ^25^, ^27^, ^29^, ^32^  The combined effort of patients and providers to create and influence new policies is notably beneficial. Perry et al.^25^ addressed that a large interest has been placed on policy creation through a collaborative model with users of healthcare. Further, Scammell et al.^27^ indicated that in places like the UK nursing policy development often involves engagement with healthcare users for advisement and recommendations.

#### 3.6.6 Decreased healthcare disparities

Five articles reported the potential of public participation in healthcare students' education to help decrease healthcare disparities.^15^, ^16^, ^18^, ^19^, ^32^ Dijk et al.^14^ highlighted that Indigenous patient populations, transgender health and cancer disparities were better understood because of patient engagement in education.^14^ Notably, the participation of mental health patients in the education of healthcare students helped challenge stigma and discrimination, thus promoting social inclusion and positive understandings.^32^

### 3.7 Considerations for curriculum

Of the 20 articles included in this review, 12 reported on future curriculum considerations for public participation in healthcare students' education.

#### 3.7.1 Integrating patient perspectives into education

Nine articles suggested the need to further integrate patient perspectives into healthcare students' education.^4^, ^14^, ^15^, ^16^, ^18^, ^19^, ^20^, ^21^, ^32^ Integration of patient narratives and perspectives into students’ education can advance student learning.^14^  For example, involving patients' lived experiences in mental health nursing education facilitated a new level of understanding for students.^20^ However, studies have identified the need for future research to determine the theoretical mechanisms through which patient involvement promotes learning.^31^ Another way in which patient perspectives may be integrated into education is the involvement of patients in learner assessment. However, some barriers may influence such involvement, including language and reading comprehension challenges and the nonreadiness of educational programmes to partner with patients.^19^ To benefit from patient perspectives in education, such barriers require addressing.

#### 3.7.2 Embedding consumer‐led initiatives into the curriculum

Nine articles highlighted the potential benefits of embedding consumer‐led initiatives into the curriculum.^4^, ^14^, ^15^, ^16^, ^18^, ^19^, ^20^, ^21^, ^32^ Consumer participation across the design, planning and delivery stages of education, including serving as a learning resource, being a collaborator, assessing learning or helping with the development of teaching materials, with patients objecting to voyeurism and tokenism.^32^ Finch et al.^16^ found that when patients give feedback on students' clinical skills it has a positive impact on learning.

#### 3.7.3 Incorporating real‐life case studies into learning

Nine articles suggested that real‐life case studies based on patient experience be incorporated into healthcare students' education.^15^, ^16^, ^18^, ^22^, ^23^, ^24^, ^32^ Dijk et al.^15^ explained that while simulations or practice with an inanimate body is useful, practice on real patients often results in more authenticity in care and provides better guidance with skills such as physical examinations. McCray et al.^23^ reported on the positive impact on student academic performance through the incorporation of student‐run clinic experiences in medical education. The use of patients as simulated patients has been reported in various healthcare professions education contexts such as with student pharmacists.^24^

#### 3.7.4 Utilizing patient‐centred teaching strategies

Six articles reported on the potential of utilizing patient‐centred teaching strategies when teaching healthcare students.^4^, ^14^, ^15^, ^16^, ^19^, ^20^ The rationale of exposing students to patient voices is to support the development of patient‐centred professional identity and the understanding of patient‐centredness.^14^, ^15^ Gordon et al.^4^ took an in‐depth look into the outcomes of utilizing teaching strategies that involve putting the patient first and suggested benefits to learners, such as a greater understanding of holistic and patient‐centred care. Khalife et al.^20^ advocated for a competency‐based medical education framework that mandates students be exposed to patient‐centred education to prepare them to meet the patient's needs.

#### 3.7.5 Promoting patient advocacy and professionalism

The promotion of patient advocacy as a part of professionalism was present in three articles.^16^, ^20^, ^32^ Arblaster et al.^32^ reported that personal contact of healthcare students with mental health patients under power‐equalizing conditions was key to stigma reduction and advocating for this patient population. Khalife et al.^20^ reported that patient involvement in assessment approaches in competency‐based education could enhance learner advocacy competencies.

#### 3.7.6 Incorporating patients as teachers

The concept of incorporating patients as teachers was present in one article.^4^ Gordon et al.^4^ highlighted that the involvement of patients as educators benefited students, including increased confidence and comfort working with patients; it also benefited patients as they felt they made a valuable and meaningful impact on healthcare. Further, Gordon et al.^4^ stated that using patients as teachers is most effective when standardized assessment checklists and scoring criteria are clear.

#### 3.7.7 Including recovery‐oriented practices

Our review identified recovery‐oriented practices in two articles.^18^, ^32^ Arblaster et al.^32^ evaluated evidence regarding the effectiveness of patient participation in producing graduates with the ability for recovery‐oriented occupational therapy practice; although the authors cautioned that minimal evidence currently exists, they highlighted that patient participation is a way to exemplify recovery‐oriented practice and that education should continue to involve patients. Happell et al.^18^ provide similar findings as they suggested that the consistent involvement of educators and patients within healthcare students' education allows students to gain a better understanding of multiple ways and paths of recovery.

#### 3.7.8 Enhancing cultural competence

Six articles reported the importance of enhancing cultural competence in healthcare students' education.^4^, ^15^, ^18^, ^19^, ^21^, ^32^ The enhancement of cultural competencies has played a more vital role in recent years as healthcare and several other industries strive to diversify and understand diversity more readily. Dijk et al.^15^ stated that one of the rationales for involving patients in medical education is the multicultural learning environment it produces that allows students to practice social accountability and inclusion.

#### 3.7.9 Increasing communication and teamwork

Seven articles suggested the need for and importance of increasing communication and teamwork within healthcare students' education.^4^, ^14^, ^15^, ^18^, ^19^ Gordon et al.^4^ provide evidence that communication and teamwork are interlinked and a vital piece of preparing both students and healthcare users to be present within clinical spaces. Skills such as professionalism, communication, patient‐centredness and holistic care can effectively be learned when patients and students communicate and work together as a team as part of educational interventions.^4^

## 4 DISCUSSION

In conducting this umbrella review we aimed to identify current, relevant and robust evidence on the impacts of public participation in healthcare students; education. Given the educational lens of our research question, it is unsurprising that all reviews we synthesized demonstrated an impact on healthcare student learning. Specifically, increased awareness of medical conditions and their effects on actual patients improved patient‐centred communication, invigorated motivation and engagement in their own training and increased empathy. Empathy is considered a core skill for most health professionals and can enhance therapeutic communication, improve patient health outcomes^33^ and influence health professional wellbeing.^34^ Unfortunately, research shows waning empathy amongst health professorial students as their education progresses^34^ and how empathy is not always enacted by health professionals.^33^ Additionally, there remains no consensus on which of the mechanisms used and studied for empathy education are most appropriate or effective.^35^ Our umbrella review has uncovered that while empathy teaching was not the original focus of these individual reviews, patient involvement in health professional education is a powerful mechanism to enhance empathy skills necessary for practice.

Public members who participated in student training identified greater satisfaction with care and increased understanding of their condition and treatment. Participation in healthcare student education fostered more trusting relationships with healthcare professionals with additional observed ripple effects on self‐advocacy and self‐management. Kangasjarvi et al.^36^ interviewed patients as teachers to understand more about their experiences participating in healthcare student education and noted that patients felt ‘re‐humanized’ with a new sense of empowerment to act for system changes. Patients have also emphasized how future graduates must spend even more time building trust with patients with increased transparency in treatment decision‐making.^37^

How public participation in healthcare education ultimately exerts long‐term impacts on healthcare and associated systems is difficult to clearly discern; such downstream and sustained effects are subject to multiple shifting influences.^38^ Nevertheless, our findings indicate changes in patient‐centred treatment decision‐making and patient safety are achievable through these educational initiatives and partnerships.

Integration of patient experiences into healthcare students' education promoted student development of skills necessary for patient care, including favourable professional attitudes and behaviours. Educators are increasingly examining how optimal conditions for public participation can be created, including addressing public member ideas for their own instructional professional development.^3^

Given the stated expectations of many health professional programmes to train graduates equipped to serve society, our findings of public participation are encouraging, but these opportunities must be maintained, expanded, and adopted by more education programmes across health professional disciplines. Most experiences reported in our review are amongst physicians and nurses in training. These students represent a large, but incomplete membership of the healthcare workforce who ultimately interface with the public. Robust evaluation plans and strategies to overcome resource constraints associated with public participation in the curriculum are needed.^1^

### 4.1 Strengths and limitations

To the best of our knowledge, this is the first umbrella review to synthesize the impact of public participation in healthcare students' education on students, the public, healthcare systems and curricula. While a systematic and comprehensive search was conducted, we limited our search to English language publications that were published in the last 10 years; therefore, we may have failed to capture some key literature relevant to this review. The heterogeneity of review types, disciplines and reported outcomes made it difficult to synthesize review findings in a generalizable manner. We chose to purposefully include reviews conducted across healthcare professions noting that the findings may not be as easily transferable to specific contexts. However, as healthcare professions are increasingly relying on public participation in healthcare education, a review that synthesizes evidence across disciplines has a unique value in terms of the transferability of knowledge.

### 4.2 Recommendations for future research

We encourage future research across healthcare disciplines to identify more unified platforms to facilitate public participation in healthcare students' education. A clear definition of public involvement in healthcare students' education is needed to gain conceptual clarity of what this entails (such as patient vs. public vs. consumer). Future research to explore and evaluate additional methods of integrating patient and public participation in healthcare students' education is warranted. Further, longitudinal research is needed to evaluate the persistence of impacts on students, patients/public and healthcare systems.

## 5 CONCLUSION

Public participation in healthcare students' education has positive impacts on students, the public, curricula and healthcare systems. Findings from this review offer critical insight for healthcare educators to consider avenues for greater collaboration amongst students, patients and the public.

## AUTHOR CONTRIBUTIONS

**Lorelli Nowell**: Conceptualization; investigation; writing—original draft; methodology; validation; visualization; writing—review and editing; formal analysis; project administration; supervision; resources. **Bryn Keogh**: Writing—original draft; formal analysis. **Eleftheria Laios**: investigation; writing—original draft; validation; conceptualization; formal analysis. **Lisa Mckendrick‐Calder**: Conceptualization; investigation; writing—original draft; validation; formal analysis. **Whitney Lucas Molitor**: Conceptualization; investigation; writing—original draft; validation; formal analysis. **Kerry Wilbur**: Conceptualization; investigation; validation; formal analysis.

## CONFLICT OF INTEREST STATEMENT

The authors declare no conflict of interest.

## Supporting information

Supporting information.

## Data Availability

Data sharing is not applicable to this article as no new data were created or analyzed in this study.
