# Supplementary material for: Public participation in healthcare students' education: An umbrella review
Source: Health Expect. 2024 Jan 21;27(1):e13974. doi: 10.1111/hex.13974 (PMC10801288; doi:10.1111/hex.13974)
Supplement: Supplementary file 1 — Supporting information. [file HEX-27-e13974-s001.docx]

**Supplementary Table 1**

**Databases**

MEDLINE, EMBASE, ERIC, PsychINFO, CINAHL, PubMed, JBI Database of Systematic Reviews and Implementation Reports, the Cochrane Database of Systematic Reviews, Database of Abstracts of Reviews of Effects and the PROSPERO register. **(limit to English and published since 2012)**

**Search Strategy**

| **Patient/public Involvement in Education** | **Type of involvement** | **Target audience of PPI in education** | **Types of articles -Reviews** |
| --- | --- | --- | --- |
| **Medical Subject Headings (MeSH)**  **(“exp” indicates exploded MeSH)** | | | |
| exp Stakeholder Participation/ *OR*  exp Patient Participation/ *OR*  exp Community Participation/ | exp Teaching/ *OR*  exp Formative Feedback/ OR  exp Simulation Training/ *OR*  exp Curriculum/ *OR*  exp Program Evaluation/ *OR*  exp Advisory Committees/ | exp Students, Health Occupations/ *OR*  exp Education, professionals/  *OR*  exp Medicine/ *OR*  exp Nursing/ *OR* exp Social Work/ *OR*  exp Midwifery/ *OR*  Language Therapy/*OR*  Speech Therapy/ *OR*  Allied Health Professionals/ *OR*  exp Occupational Therapy/ *OR*  exp Pharmacists/ *OR*  exp Dentists/ *OR*  exp Clinical Psychology/ | exp review/OR  exp literature review/ OR exp meta-analysis/OR  systematic/ OR  Meta-synthesis |
| **Free Text Search Terms** | | | |
| “Patient Public Involvement”.mp *OR*  (Patient* involv*” or “Patient* collaborat*” or “Patient* Participat*” or “Patient* integrat*” or “Patient* engag*” or “Patient* partner*” or “Patient* co-produc*”).mp *OR*  (“Carer* involv*” or “Carer* collaborat*” or “Carer* participat*” or “carer* integrat*” or “Carer* engag*” or “Carer* partner*” or “Carer* Co-produc*”).mp *OR*  (“Client* involv*” or “Client* collaborat*” or “Client* participat*” or “Client* integrat*” or “Client* engag*” or “Client* partner*” or “Client* co-produc*”).mp *OR*  (“Service user* involv*” or “Service user* collaborat*” or “Service user* participat*” or “Service user* integrat*” or “Service user* engag*” or “Service user* partner*” or “Service user* co-produc*”).mp *OR*  (“Consumer* involv*” or “consumer* collaborat*” or “Consumer* participat*” or “Consumer* integrat*” or “Consumer* engag*” or “Consumer* partner*” or “Consumer*co-produc*”).mp  *OR*  (“Patient teacher*” or “Patient educator*” or “patient led teaching” or “Patient instructor*” or “Patient mentor*”).mp | (teaching or learn* or training or assess* or “story-tell*” or feedback or simulat*) *OR*  ((course* or module* or curricul* or program*) AND (deliver* or design* or develop* or evaluat*)) | (Medic* or doctor* or nurs* or “social work*” or “social care” or midwif* or “speech and language therap*” or pharmac* or dentist* or “allied health” or physiotherapy* or “mental health nurs*” or “occupational therap*” or “health professional*” or “healthcare professional*” or interprofessional* or “multi professional*” or “clinical psychology*”).mp  *AND*  (educat* or undergraduate* or postgraduate* or student* or learner* or trainee* or “graduate entry” or graduate* or bachelors or “pre-registration” .mp | Literature review or review or meta-analysis or systematic or meta-synthesis |
